# Comparison of food intake pattern of diabetic patients and healthy individuals in a sample of Saudi population: a case-control study

**DOI:** 10.1186/s12889-024-19064-x

**Published:** 2024-06-13

**Authors:** Afnan H. Saaty, Haya MA. Aljadani

**Affiliations:** Food and Nutrition Department, Faculty of Human Sciences and Design, King Abdulaziz University, Jeddah, 21551 Saudi Arabia

**Keywords:** Type 2 diabetes, Food consumption pattern, Dietary behavior, Physical activity, Diabetes risk factors

## Abstract

**Background:**

There has been a significant rise in the number of individuals diagnosed with type 2 diabetes mellitus (T2DM), with the condition reaching epidemic proportions globally. This study examined the dietary pattern of a sample of Saudi Arabian adults with T2DM compared to control non-diabetics.

**Methods:**

Data from 414 participants, 207 control and 207 T2DM was analyzed. Anthropometric measurements, foods intake such as vegetables, fruits, whole grains, fried foods, sweetened juice, sweets, and pastries consumption as well as physical activity were obtained by an interview-survey.

**Results:**

The consumption of vegetables, green and leafy vegetables, starchy vegetables, fruits, proteins, and milk was significantly higher in the diabetics (p< 0.0001 for all and p<0.01 for starchy vegetables). Of the case group, 79.7% of them consumed whole-wheat bread while 54.6% of them consumed low fat milk (p<0.0001). There was a significant decrease in the percentage of cases who consumed discretionary foods and sweetened juices and soft drinks (24.1%), avoided sweets (75.8%) and pastries (37.1%), (p<0.0001). There were also significant increases in the percentages of participants who use healthy fat (as olive oil) in the case group (78.7%) (p<0.001). There was a significant increase in the percentage of diabetics who followed a diet to lose weight (15%) (p<0.05). The majority of the two study groups were physically inactive (control 95.2% & case 94.2%).

**Conclusions:**

The results of this study provide insight on that diabetics generally follow a healthy diet, yet their engagement in physical activity may not be optimal.

## Introduction

Nutrition plays a critical role in the maintenance of good health and the prevention of disease. The burden of nutrition-related chronic diseases is elevating rapidly worldwide [1]. Unhealthy diets are a major culprit behind the world’s disease burden and deaths, according to extensive research from the Global Burden of Disease Study [2]. Nowadays, diabetes is one of the most predominant major public health chronic diseases due to its growing epidemic prevalence. It is one of the top ten causes of death worldwide [3]. Globally, it is estimated that 10.5% of adults aged 20–79 years were diagnosed with type 2 diabetes mellitus (T2DM) in 2021. This number is forecast to grow to 12.2% by 2045 [4]. One in five people in the Gulf Cooperation Council region has T2DM, which is amongst the highest prevalence worldwide. With an average frequency of 15%, Saudi Arabia ranks among the top 20 nations with the highest rates of diabetes per capita [5].

Nutrition and lifestyle practices are acknowledged as integral components of successful T2DM management plans in improving patients’ clinical outcomes and quality of life [6]. In the past ten years, there has been a rise in various dietary strategies aimed at aiding individuals with T2DM. These alternative approaches to national dietary guidelines encompass a range of dietary patterns, such as the Dietary Approaches to Stop Hypertension (DASH), intermittent fasting, low-carbohydrate, low-fat, low-glycemic index, Mediterranean, and plant-based options [7–9]. Healthy dietary practices effectively reduce hemoglobin A1c (HbA1c) levels in T2DM patients and improve glucose tolerance, blood pressure, lipid profile, and the onset of diabetes complications, thus resulting in declining glucose-lowering medication doses. They were, besides, shown to be vital in preventing the progression of prediabetes into T2DM [10].

The development of T2DM is multifaceted, involving both irreversible factors like age, genetics, race, and ethnicity, as well as reversible factors such as diet, physical activity, and smoking [11]. The consumption of simple and refined carbohydrates, overeating, a sedentary lifestyle, and smoking influence the development of diabetes. Poor dietary patterns including the increased ingestion of processed foods, saturated fats, and simple carbohydrates, with decreased dietary fiber intake elevate the chances of T2DM [12]. It is revealed that prolonged ingestion of high glycaemic load foods boosts the chances of T2DM [13].

Monitored dietary intake and lifestyle practices allow for tracking the person’s food habits, which can help healthcare professionals in Saudi Arabia provide more individualized and culturally relevant dietary advice to their diabetic patients, taking into account their specific food intake patterns. This study aimed to describe diet pattern and behavior and physical activity from a nationally representative sample of Saudi Arabian adults with T2DM compared to control non-diabetic participants. A case-control design has been chosen as it is well-suited for comparing these characteristics at a single point in time and can efficiently capture an adequate number of cases and controls in a population where T2DM is relatively prevalent.

## Materials and methods

### Study design

The case-control study described in this article was carried out in Jeddah, Saudi Arabia, between April 2021 and May 2022. It recruited 207 adults with type 2 diabetes and 207 adults non-diabetic individuals. Control individuals and cases were matched for age (*±* 5 years) and gender. Patients with T2DM were recruited randomly from the outpatient clinics of the Jeddah Care Center for Diabetics and Hypertension, Saudi Arabia. It is the only specialities government center at Jeddah that patients with diabetics from all Jeddah and the surrounding area visit to follow-up.

The control group was recruited from the general population in different sittings such as health clinics, primary care clinics and educational institutions. To ensure comparability between the control and cases, individuals were carefully screened and matched based on age, gender, and education level. Additionally, control subjects were selected based on the absence of any known medical conditions.

### Inclusion and exclusion criteria

All participants must be Saudi nationals of either gender, and older than 35. It was reported that a notable increase in the incidence of diabetes in individuals with younger age around 30s has been observed [14]. The participants in the T2DM group had T2DM for more than a year prior to their diagnosis. Individuals in this group must have a confirmed diagnosis of type 2 diabetes according to established diagnostic criteria (e.g., medical records, physician diagnosis). The control group included those with no incident of T2DM or type one diabetes. Exclusion criteria included those who were pregnant or breast feeding if women, and if they have type one diabetes because having such condition will affect their food habits. Participants who did not meet the inclusion criteria were excluded. Prior to the start of the study, participants provided informed consent agreeing to the required measurement and survey completion procedures. The Study protocol complied with the University research ethic committee and the principles embodied in the Helsinki declaration and the ethical approval for the study was obtained from the Ministry of Health: Number (A01911).

### Data collection

A developed questionnaire was used in the study to collect data from the participants during an interview. This was tested and validated by performing a pilot study involving a sample of 20 participants to evaluate the survey instrument, and based on the feedback received, necessary modifications were made to improve its validity and reliability. Subsequently, the final version of the survey underwent an evaluation process by five independent experts in nutrition from King Abdul Aziz University, Saudi Arabia. These experts carefully reviewed the survey and provided their suggestion to ensure that the questions were appropriate and avoided any misunderstanding in order to answer them in the intended way. The data were obtained *via* face-to-face interviewing. The questionnaire allowed researchers to obtain information about demographics, health status, physical activity, food intake, and dietary behaviors that related to maintaining blood glucose.

The questionnaire consisted of a total 24 questions classified into three main sections. The initial section of the questionnaire included information about demographic, health, and physical activity. Health status assessed the diabetes complications for T2DM participants. From those with T2DM. Data related to HbA1c were taken from the patient’s file. To measure physical activity, it adopted question from American College Health Association National College Health Assessment (ACHA-NCHA) survey [15].

Three qualified nursing were actively involved in the precise measurement of both weight and height parameters. Each nurse, possessed expertise in anthropocentric measurements, followed established scientific procedures during the assessments. A balance beam scale was used to measure a subject weight and height by a trained dietician. When measuring weight, a subject was asked to take off the shoes and any heavy items such as bags, phone and was with ideally clothing. A subject asked to stand still and stretch upward and vertex of client’s head was measured. Then BMI were calculated for each subject and grouped into those with healthy weight.

To gather information about an individual’s dietary intake, participants were asked about their consumption of certain food groups according to the US Myplate. The questionnaire asks about the usual frequency of food consumption of the last week. This study focused on the vegetable group and divided them according to the US Myplate into dark-green vegetables (raw leafy greens, lettuce, broccoli… etc.), red and orange vegetables (red and oranges bell paper, tomatoes carrot…etc), starchy vegetables (green peas, corn, potatoes), and other vegetables that include okra, cucumber, onion and others. In addition, food groups included fruit, meat and alternatives, bread and grains, as well as information on the types of milk or dairy products consumed. For each food group, participants selected the frequency of their intake of the food. They were also asked to choose the estimated portion size of a given food group each time they would consume [16–18]. Participants have to respond to the following questions: How many serving of fruit and vegetables do they consume per day on average? (On the basis that one serving of vegetables is equal to one carrot or other fresh vegetable, one small bowl of green salad, or cooked veggies). One serving of fresh fruit, such an apple, or a small bowl of fruit salad. The range of response possibilities for the dietary factors was 0 to more than 4 servings. Additionally, the type of veggies consumed was requested (green leafy vegetables, red and orange vegetables, and starchy vegetables). They were also asked about the variety of vegetable intake. In addition, the participants were asked regarding their typical intake of various dietary groups, such as protein (meat, fish, and eggs), grains (rice, bread, and pasta). The serving size for each food item in each question is fully described for each group (as one serving of protein equal to one egg of a piece of cooked lean meat or poultry is about the size of a deck of cards or the palm of their hand). The type of milk and bread the participants consumed was also questioned.

The third section of the questionnaire assessed the participants’ dietary behaviors, including consumption of discretionary foods and drinks including sweets, pastries, fried food, fat, sweetened juices, and soft drinks. There are other questions related to using sugar substitutes (artificial sweeteners), whole wheat bread, consuming unsaturated fat such as olive oil or saturated fat, cooking lean meat, and cooking chicken without the skin. Also, they were asked if they follow a low-carb diet or other diets to lose weight. The prediabetes risks score for participants has been calculated based on the tool available on the Saudi Ministry of Health’s website, assigning points based on responses to specific questions. If the total score is 4 or lower, it suggests a lower risk of pre-diabetes, and individuals are encouraged to maintain a healthy lifestyle for overall well-being. If the total score is 5 or higher, it indicates a higher susceptibility to pre-diabetes and, consequently, type 2 diabetes [19].

### Statistical calculations

The collected data underwent a descriptive analysis. Frequency of number (n) and percentage (%) were used to depict the data. The chi-square test and unpaired student t-test were used for data analysis. For the statistical analysis, GraphPad software version 8.4.0 (Inc., La Jolla, CA, USA) was utilized. The significance level was chosen at p<0.05.

## Results

### Demographic and general characteristics of diabetic and control participants

A total of 414 participants were recruited in the study. They were divided into two groups, the control group (*n* = 207) and the diabetes group (*n* = 207). The average ages of participants was 47.6 years. Table 1 showed the demographic and general characteristics of the study groups. There was a significant difference between the control group and the diabetes group concerning average BMI (control 28.5 *±* 0.4 & diabetes 30.2 *±* 0.5) (p<0.01). The over-weight participants constituted the majority of the control group (42.5%) while the obese participants constituted the majority of the diabetes group (47.8%). About 82.6% of the control group were married compared to 64.7% of the diabetes group (p<0.0001). Highly educated participants were most prevalent in the control (85%) and diabetes (66%) groups, with a significant difference of less than 0.0001. of the control group, 63% were employees compared to 48.5% of the diabetes group (p<0.01). High monthly income participants constituted both the study groups (control 82.9% & diabetes 89.2%). The majority of the two groups were non-smokers (control 89.9% & diabetes 79.2%), while smokers constituted 20.7% of the diabetes group compared to only 10.1% of the control group (p<0.01). Furthermore, most of the two study groups were physically inactive (control 94.2% & diabetes 95.2%). Concerning the family history of diabetes, there was a significant difference between percentages of participants who reported they had a family history of diabetes in the diabetes group (93.7%) compared to the control group (87.4%) (p<0.05). Of the control group, 53.1% had prediabetes risk factors. Hyperlipidemia and hypertension presented in 58.8% and 53.4% of the diabetes group compared to 10% and 5.2% of the control group (p<0.0001). The average HbA1c value of the diabetes group was 7.9. Concerning diabetes complications, 23% of the diabetes group reported retinopathy, 11.3% had nephropathy, and 19.1% had diabetic foot.

**Table 1:** Demographic and general characteristics of diabetic and control participants

	**Control group**	Diabetes^××^ group	*p value*
	**(n = 207)**	**(n = 207)**	
	n (%)	n (%)	
**BMI (Mean ± SD)**	28.5 ± 0.4	30.2 ± 0.52	0.0084^^^
**Under weight (BMI<18.5)**	3 (1.5%)	0 (0.0%)	0.0062^*^
**Normal weight (BMI 18.5-<25)**	51 (24.6%)	38 (18.4%)	
**Over weight (BMI 25-<30)**	88 (42.5%)	70 (33.8%)	
**Obese (BMI ≥ 30)**	67 (32.4%)	99 (47.8%)	
**Educational status**			
University and higher	176 (85.0%)	138 (66.7%)	< 0.0001^*^
Secondary and lower	31 (15.0%)	69 (33.3%)	
**Employment status**			
No	76 (37.0%)	106 (51.5%)	0.0031^*^
Yes	131 (63.0%)	101 (48.5%)	
**Marital status**			
No	36 (17.4%)	73 (35.3%)	< 0.0001^*^
Yes	171 (82.6%)	134 (64.7%)	
**Personal monthly income**			
Low	34 (16.4%)	23 (11.1%)	0.0676
High	173 (83.6%)	184 (88.9%)	
**Smoking**			
No	186 (89.9%)	164 (79.2%)	0.0066^*^
Yes	21 (10.1%)	43 (20.7%)	
**HbA1c (Mean ± SD)**	-	7.9 ± 0.09	
**Family history of diabetes**			
No	26 (12.6%)	13 (6.3%)	0.0108^*^
Yes	181 (87.4%)	194 (93.7%)	
**Prediabetes risks**			
No	97 (46.9%)	-	
Yes	110 (53.1%)		
**Comorbidities**			
Hyperlipidemia	21 (10.0%)	120 (58.8%)	< 0.0001^*^
Hypertension	11 (5.2%)	109 (53.4%)	< 0.0001^*^
**Diabetes complications**			
Retinopathy	-	47 (23.0%)	
Nephropathy	-	23 (11.3%)	
Diabetic foot	-	39 (19.1%)	
**Physical activity**			
Active (5–7 times/week)	12 (5.8%)	10 (4.8%)	0.3929 ^*^
Not active (≤ 1–4 times/week)	195 (94.2%)	197 (95.2%)	

Note: ^*^chi-square; ^^^unpaired student t test; ^**××**^type 2 diabetes; SD: standard deviation, n: number; %: percentage related to sample size; *p values* are two-sided

### Comparison of food intake between diabetic and control participants

Table 2 showed the food intake of the diabetic and control participants. The frequency of vegetables intake and the consumption of total vegetables, dark green vegetables, starchy vegetables, and other vegetables was significantly higher in the diabetes group than in the control group (p<0.0001, 0.0001, 0.0001, 0.01, and 0.01, respectively). Of the diabetes group, 46.4% of the participants consumed vegeTables 2, 3 and 4 times daily compared to 12.6% of the control group participants. Also, 17.4% of the people with diabetes consumed vegetables more than 4 times daily compared to 4.3% of the control participants. Of the diabetes group, 51.2% of the participants consumed more variety of vegetables > 4 items/ week compared to 15.1% of the control group participants. Approximately 36.2% of the people with diabetes consumed dark green vegeTables 2, 3 and 4 serving daily in their diet compared to 10.6% of the control participants. About 17.4% of the people with diabetes consumed starchy vegeTables 2, 3 and 4 serving daily compared to 5.8% of the control participants.

The consumption of fruits, proteins, and milk was significantly higher in the diabetes group than in the control group (p<0.0001). Of the diabetes group, 29.5% of the participants consumed fruits 2–4 serving daily in their diet compared to 9.6% of the control group participants. Protein intake was lower in diabetes patients (65.7%) compared to 86% of the control participants. Nearly 26% of the diabetics’ consumed milk and dairy products 2–4 serving daily compared to 9.2% of the control participants.

The Cases with diabetes (79.7%) appeared more likely to consume whole-wheat bread, rather than refined bread in their diet, compared to 48.3% of the control group participants (p<0.0001). Consumption of full-fat milk was lower in the diabetes patients (35.3%) compared to 55% of the control participants (p<0.0001). While 54.6% of the diabetics consumed low-fat milk compared to 35.3% of the control participants (p<0.0001).

There were no significant differences between the daily consumption of red and orange vegetables and grain among the diabetics and the control participants.

**Table 2:** Comparison of food consumption pattern between diabetic and control participants

	Control group	Diabetes^××^ group	*p value*
	(n = 207)	(n = 207)	
	n (%)	n (%)	
**Vegetables**			
**Frequency**			
≤ 1 time/day	172 (83.1%)	75 (36.2%)	< 0.0001^*^
2–4 time/day	26 (12.6%)	96 (46.4%)	
> 4 times/day	9 (4.3%)	36 (17.4%)	
**Total vegetables**			
< 3 item/week	111 (53.6%)	50 (24.2%)	< 0.0001^*^
3–4 item/week	65 (31.4%)	51 (24.6%)	
> 4 item/week	31 (15.0%)	106 (51.2%)	
**Dark green vegetables**			
≤ 1 serving/day	182 (88.0%)	128 (61.8%)	
2–4 serving/day	22 (10.6%)	75 (36.2%)	< 0.0001^*^
> 4 serving/day	3 (1.4%)	4 (2.0%)	
**Red and orange vegetables**			
≤ 1 serving/day	201 (97.1%)	197 (95.1%)	
2–4 serving/day	5 (2.4%)	8 (3.9%)	0.8126^*^
> 4 serving/day	1 (0.5%)	2 (1.0%)	
**Starchy vegetables**			
≤ 1 serving/day	194 (93.7%)	168 (81.1%)	0.0031^*^
2–4 serving/day	12 (5.8%)	36 (17.4%)	
> 4 serving/day	1 (0.5%)	3 (1.5%)	
**Other vegetables**			
≤ 1 serving/day	197 (95.2%)	170 (82.2%)	0.0002^*^
2–4 serving/day	9 (4.3%)	37 (17.8%)	
> 4 serving/day	1 (0.5%)	0 (0.0%)	
**Fruits**			
≤ 1 serving/day	186 (89.9%)	146 (70.5%)	< 0.0001^*^
2–4 serving/day	20 (9.6%)	61 (29.5%)	
> 4 serving/day	1 (0.5%)	0 (0.0%)	
**Proteins**			
Never	5 (2.4%)	1 (0.5%)	< 0.0001^*^
≤ 1 serving/day	178 (86.0%)	136 (65.7%)	
2–3 serving/day	24 (11.6%)	70 (33.8%)	
**Grain**			
≤ 5 serving/day	202 (97.6%)	204 (99.0%)	0.4496^*^
> 5 serving/day	5 (2.4%)	3 (1.0%)	
**Type of bread**			
Whole-wheat	100 (48.3%)	165 (79.7%)	< 0.0001^*^
White	69 (33.3%)	32 (15.4%)	
Mix	21 (10.2%)	4 (2.0%)	
Other	17 (8.2%)	6 (2.9%)	
**Milk and dairy products**			
≤ 1 serving/day	188 (90.8%)	153 (74.0%)	0.0001^*^
2–4 serving/day	19 (9.2%)	54 (26.0%)	
**Type of milk**			
Full fat milk	114 (55.0)	73 (35.3%)	< 0.0001^*^
Low fat milk	73 (35.3%)	113 (54.6%)	
Skimmed milk	4 (2.0%)	0 (0.0%)	
No milk	16 (7.7%)	21 (10.1%)	

### Comparison of dietary behaviors between diabetic and control participants

Table 3 showed the dietary behaviors of diabetic and control participants. There was a significant decrease in the percentage of diabetics consuming sweetened juices or soft drinks (24.1%) compared to the control group (48.3%) (p<0.001). On the other hand, there were significant increases in the percentage of diabetics consuming sugar substitutes (artificial sweeteners) 48.1% compared to the control group 3.8% (p<0.0001). There were significant increases in the percentages of participants who avoid discretionary food such as sweets, pastries, and fried food consumption in the diabetes group (75.8%, 37.1%, and 45.8%, respectively) compared to the control group (8.2%, 6.2%, and 7.7%, respectively) (p<0.0001). There were also significant increases in the percentages of participants who cooked lean meat and who used healthy fat such as olive oil instead of saturated fat such as butter in the diabetes group (66.2% & 78.7%) compared to the control group (51.7% & 55%) (p<0.001 & 0.0001, respectively). There were significant increases in the percentages of participants who cooked chicken without the skin and who reduced fat consumption in the control group (55% & 58.9%) compared to the diabetes group (40.5% & 44.4%) (p<0.01). There was a significant increase in the percentage of people with diabetes who followed a diet to lose weight (14.9%) compared to the control group (7.7%) (p<0.05). There was no significant difference between control and diabetic participants who followed a low-carb diet.

**Table 3:** Comparison of dietary behaviors between diabetic and control participants

	Control group (*n* = 207) *n* (%)	Diabetes^××^ group (*n* = 207) *n* (%)	*p* value
Consumption of sweetened juices or soft drinks	100 (48.3%)	50 (24.1%)	0.0001^*^
Use sugar substitutes	8 (3.8%)	100 (48.1%)	< 0.0001^*^
Use whole-wheat bread	100 (48.3%)	165 (79.7%)	< 0.0001^*^
Avoid sweets	17 (8.2%)	157 (75.8%)	< 0.0001^*^
Avoid pastries	13 (6.2%)	77 (37.1%)	< 0.0001^*^
Avoid fried food	16 (7.7%)	95 (45.8%)	< 0.0001^*^
Cooking lean meat	107 (51.7%)	137 (66.2%)	0.0007^*^
Cooking chicken without the skin	114 (55.0%)	84 (40.5%)	0.0106^*^
Reduce fat consumption	122 (58.9%)	92 (44.4%)	0.0107^*^
Use healthy fat	114 (55.0%)	163 (78.7%)	< 0.0001^*^
Follow low carb diet	11 (5.3%)	16 (7.7%)	0.3224^*^
Follow a diet to lose weight	16 (7.7%)	31 (14.9%)	0.0194^*^

Note: ^*^chi-square; ^**××**^type 2 diabetes; n: number; %: percentage related to sample size; *p values* are two-sided

## Discussion

Despite the importance of regulating dietary factors for effective diabetes management and the presence of Saudi guidelines regarding diabetic diets, little is still known about the actual dietary patterns followed by diabetic individuals in Saudi Arabia. Diabetic patients in this study consumed significantly more vegetables, fruits, protein, and milk compared to the healthy individuals. They also showed a preference for whole-wheat bread and low-fat milk, while significantly reducing their intake of sugary drinks, sweets, and pastries. Additionally, a higher percentage of diabetics used healthy fats like olive oil and followed weight-loss diets.

The comparison between the consumption of food groups between healthy participants and patients with T2DM revealed significant differences in most food groups. Compared to the control participants, diabetics consumed a diet high in fruits, protein, dairy and low-fat dairy products, whole-wheat bread, and healthy fats from vegetable oils like olive oil. Diabetics also consumed a variety of green, fibrous, and starchy vegetables. This demonstrated that, compared to the healthy participants, diabetics led a healthier lifestyle. To reduce complications of diabetes, treatment techniques usually include dietary and lifestyle changes and increased physical activity [20]. In their narrative review, Hallberg et al. observed that there is data suggesting that T2DM may be reversed using many strategies, including bariatric surgery, low-calorie diets, or carbohydrate restriction. In particular, a low-carb diet and short-term low-calorie diets are endorsed by the American Diabetes Association and the European Association for the Study of Diabetes [21].

Three or more servings of vegetables per day were inversely related to T2DM [22]. Antioxidants like polyphenols, carotenoids, and vitamin C, which are abundant in fruits and vegetables, have been linked to a lower incidence of T2DM [23, 24]. According to Wang et al. meta-analysis, eating more raw fruits and vegetables, mainly green leafy, yellow, and cruciferous vegetables, has the potential to reduce the chance of developing T2DM [25]. Additionally, there are claims that eating green, leafy vegetables lowers the chance of getting T2DM [26]. However, a different prospective study revealed no link between eating vegetables and the incidence of T2DM [27]. According to other research, consuming either fruits, solely vegetables, or a combination of both did not significantly decrease the risk of T2DM [26, 28].

Among the most frequent lipid disorders shown in clinical practice is hypertriglyceridemia. The most frequent causes of hypertriglyceridemia are unmanaged diabetes and fatness [29]. A study discovered that individuals who consumed at least two servings of fruits and vegetables daily had reduced probabilities of developing hypertriglyceridemia than the general population, as well as lower levels of total cholesterol in women [30]. These investigations may therefore corroborate our findings that T2DM people may be trying to manage their blood lipids and diabetic states by consuming a lot of vegetables.

Most of the diabetic and control participants in this study ate less than one serving of protein daily. Dietary recommendations dictate not to exceed one serving (100–150 g) per day of any food group that includes meat, fish and eggs. The American Diabetes Association’s dietary recommendations for managing adults with diabetes stresses that the recommended amounts of protein for diabetics do not vary markedly from the proposals for the overall population [31]. Protein-rich foods may help diabetics maintain weight loss and improve dietary adherence [32–34]. A meta-analysis concluded that increased red and processed meat consumption was associated with an increased risk of T2DM [35]. Increased protein ingestion from vegetable sources (legumes) was linked to a lower chance of developing T2DM, whereas increased consumption of protein from animal origin was not (red meat) [36]. Eating meat, especially red and processed meat, greatly increases the risk of insulin resistance and non-alcoholic fatty liver disease [37]. Increased saturated fats consumption was associated with insulin resistance, impaired fasting glucose, and higher glucose concentrations on the 2-hour oral glucose tolerance test [38].

In this study, 99% of diabetics used whole grains in their diet. They reported eating no more than five servings of whole grains each day. Whole grains are supposed to be the primary source of carbs, per the recommendations [39]. Malaeb et al. found that eating whole grains is associated with significantly lower increases in postprandial glucose and insulin levels when compared to eating processed grains [40]. Consuming whole grains reduces the chance of developing prediabetes [41]. A high-fiber diet is associated with a reduced risk of T2DM [42]. In a randomized, double-blind study, people who ate bagels high in resistant starch, unlike participants who ate regular bread, had lower fasting and postprandial insulin levels and lower fasting insulin resistance [43]. Whole grains’ positive effects may be partially attributed to their high concentration of phytochemicals, vitamins, and minerals [44–46].

The result of the present paper showed that discretionary foods and sweetened juice consumption was lower in T2DM participants compared to the control participants. Fruit juices are distinguished by having less fiber and more monosaccharides than fresh fruit, which also high in glycemic index. Additionally, fruit juices are usually artificially sweetened with glucose-fructose syrup (also called high fructose corn syrup) which is less expensive than sucrose for improving the flavor of juices. In general, consuming one serving more of fruit juice per day was linked to a 7% higher risk of T2DM [47]. A meta-analysis examining the effects of soda and fruit juice consumption on T2DM found that both soda and fruit juice consumption increased the risk of diabetes [48]. Despite fructose has a reduced glycemic index compared to glucose, it is thought that its ingestion encourages the accumulation of fats in visceral abdominal region and lowers glucose tolerance and insulin sensitivity, particularly in obese people [49]. A meta-analysis of 11 cohort studies reported that participants with the highest intake of sugary drinks (1–2 drinks per day) had a 26% increased risk of developing T2DM [50]. Ingesting sugar-sweetened drinks was linked to a five times more significant risk of abdominal adiposity in diabetics, which was linked to enhanced insulin resistance and an increased risk of cardiovascular diseases [51].

There is conflicting data regarding the connections between T2DM and fried foods. According to a meta-analysis, Western dietary patterns correlate reasonably with T2DM, with up to 41% higher odds of T2DM when compared to healthy nutritional habits [52, 53]. The kind of oil used and the manner of cooking must be considered. Food that has been fried contains more calories and is more palatable, which could result in weight gain. Frying also changes the chemical makeup of the particular oils used [54]. Some fatty acids change into trans-fatty acids and potentially dangerous bioactive substances at high temperatures. They are highly prevalent in several fried foods and are well-known risk factors for T2DM and other cardiovascular diseases [55].

For the overall population and diabetics, replacing dietary sources of saturated fatty acids, such as butter and lard, with vegetable oils, which are a supplier of unsaturated fatty acids and vitamin E, is advised [56]. Olive oil is an excellent source of monounsaturated fatty acids and bioactive substances, including hydroxytyrosol and oleuropein, which may have antidiabetic and antioxidant properties [57]. According to a study, eating a diet high in virgin olive oil in the Mediterranean region may slow the course of T2DM retinopathy [58]. Data from 22 years of Nurses’ Health Research project showed that higher intake of olive oil was linked to a lower risk of acquiring T2DM in women [59].

Dairy products are a huge category with a wide range of nutritional values. The raw material of all dairy products is milk. In the meta-analysis carried out by Tong et al. [60], it was found that individuals who ingested milk and dairy products had a 14% reduced chance of developing T2DM than those who ingested the least dairy products. The same investigators concluded that there was only a negative relationship between dairy product intake and T2DM risk regarding skimmed and low-fat milk products and no such relationship has been identified for full-fat milk products. It is guessed that high levels of calcium and vitamin D reduce the risk of T2DM by regulating pancreatic beta-cell function, increasing insulin sensitivity, and affecting cytokine secretion, thereby reducing systemic inflammation [31, 61].

Similar to the findings of Basiak et al. [47] in comparison to the control participants, the diabetic participants avoided the consumption of higher glycemic indexes food groups (fruit juices, soft drinks with added sugar, sweets, pastries, fried foods, and white bread). The results may indicate that dietary advice given following a diabetes diagnosis is effective and improves the participants’ eating preferences. Evidence from the literature suggests that dietary counselling is useful in both boosting diabetes control and halting the development of full-blown symptoms of diabetes [62].

The current study has some limitations. The possibility of bias in selection exists. Those who declined to take part in the study may have characteristics that are comparable to those of the general population. Another limitation of the study is that the cases were diagnosed with T2DM more than 7 years ago, that may warrant sever complications affecting the participants’ dietary and health behaviors. Also, some participants of the case group may have modified their lifestyle behaviors, including eating and exercise routines, since they may have already learned about a healthy diet and lifestyle. Moreover, due to the limited resources, HbA1c is not tested for the control groups and only the prediabetes risk tool has been calculated. Therefore, it is plausible that some participants in the control group may have undiagnosed diabetes or prediabetes.

This study contributes to the body of knowledge by clarifying in details the different dietary practices and lifestyle of diabetes patients and non-diabetics in Jeddah, Saudi Arabia. Moreover, the design of the study was a case–control to recognize the factors linked to dietary habits and diabetes. Therefore, our results may contribute to the development of a prevention program to apply key techniques for diabetes prevention and treatment, such as improving nutritional status and changing lifestyle habits. Future research is encouraged to examine the factors that may be responsible for people’s food choices and unhealthy eating habits, as well as the importance of healthcare professional in addressing these conditions since they might result in additional diabetes illnesses and complications.

## Conclusion

The results of this study showed that T2DM participants followed a diet that avoided foods with a high glycemic index. Diabetics also consumed whole grains, whole-wheat bread, and low-fat dairy products. They avoided eating sugary drinks, sweets, pastries, and fried foods. The diabetics relayed on healthy fat sources like olive oil and followed a diet to lose weight. However, their level of involvement in physical activity was minimal. A limitation of the study is the potential for recall bias, as participants may have difficulty to accurately recall past events. Additionally, the information provided by participants regarding their beliefs about what they should be doing was not verified for accuracy or validity.

## Data Availability

Not applicable.

## Abbreviations

T2DM: Type 2 diabetes mellitus
BMI: Body Mass Index
HbA1c: Hemoglobin A1c
DASH: The Dietary Approaches to Stop Hypertension
SD: Standard deviation, n:number
%: Percentage
